# *Toxoplasma gondii* infection regulates apoptosis of host cells via miR-185/ARAF axis

**DOI:** 10.1186/s13071-023-05991-y

**Published:** 2023-10-19

**Authors:** Dingzeyang Su, Shifan Zhu, Zhaofeng Hou, Fuxing Hao, Kangzhi Xu, Fan Xu, Yuyang Zhu, Dandan Liu, Jinjun Xu, Jianping Tao

**Affiliations:** 1 College of Veterinary Medicine, Yangzhou University, 12 East Wenhui Road, Yangzhou, 225009 Jiangsu People’s Republic of China; 2 Jiangsu Co-Innovation Center for Prevention and Control of Important Animal Infectious Diseases and Zoonosis, Yangzhou, 225009 People’s Republic of China; 3 International Research Laboratory of Prevention and Control of Important Animal Infectious Diseases and Zoonotic Diseases of Jiangsu Higher Education Institutions, Yangzhou University, Yangzhou, 225009 People’s Republic of China; 4 Jiangsu Agri-Animal Husbandry Vocational College, Taizhou, 225300 People’s Republic of China

**Keywords:** *Toxoplasma gondii*, Host cell, miR-185, ARAF, Apoptosis, Regulation

## Abstract

**Background:**

Toxoplasmosis is a zoonosis with a worldwide presence that is caused by the intracellular parasite *Toxoplasma gondii*. Active regulation of apoptosis is an important immune mechanism by which host cells resist the growth of *T. gondii* or avoid excessive pathological damage induced by this parasite. Previous studies found that upregulated expression of microRNA-185 (miR-185) during *T. gondii* infection has a potential role in regulating the expression of the ARAF gene, which is reported to be associated with cell proliferation and apoptosis.

**Methods:**

The expression levels of miR-185 and the ARAF gene were evaluated by qPCR and Western blot, respectively, in mice tissues, porcine kidney epithelial cells (PK-15) and porcine alveolar macrophages (3D4/21) following infection with the *T. gondii* ToxoDB#9 and RH strains. The dual luciferase reporter assay was then used to verify the relationship between miR-185 and ARAF targets in PK-15 cells. PK-15 and 3D4/21 cell lines with stable knockout of the ARAF gene were established by CRISPR, and then the apoptosis rates of the cells following *T. gondii* infection were detected using cell flow cytometry assays. Simultaneously, the activities of cleaved caspase-3, as a key apoptosis executive protein, were detected by Western blot to evaluate the apoptosis levels of cells.

**Results:**

Infection with both the *T. gondii* ToxoDB#9 and RH strains induced an increased expression of miR-185 and a decreased expression of ARAF in mice tissues, PK-15 and 3D4/21 cells. MiR-185 mimic transfections showed a significantly negative correlation in expression levels between miR-185 and the ARAF gene. The dual luciferase reporter assay confirmed that ARAF was a target of miR-185. Functional investigation revealed that *T. gondii* infection induced the apoptosis of PK-15 and 3D4/21 cells, which could be inhibited by ARAF knockout or overexpression of miR-185. The expression levels of cleaved caspase-3 protein were significantly lower in cells with ARAF knockout than in normal cells, which were consistent with the results of the cell flow cytometry assays.

**Conclusions:**

*Toxoplasma gondii* infection could lead to the upregulation of miR-185 and the downregulation of ARAF, which was not related to the strain of *T. gondii* and the host cells. *Toxoplasma gondii* infection could regulate the apoptosis of host cells via the miR-185/ARAF axis, which represents an additional strategy used by *T. gondii* to counteract host-cell apoptosis in order to maintain survival and reproduce in the host cells.

**Graphical Abstract:**

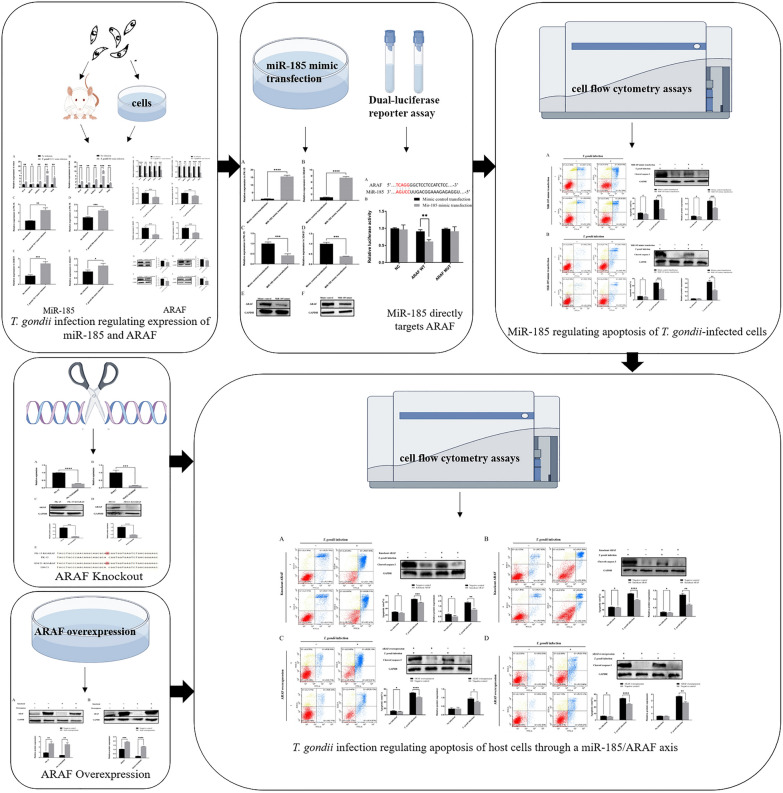

## Background

*Toxoplasma gondii* is an obligate intracellular parasitic protozoan belonging to the phylum Apicomplexa. The parasite is globally distributed and has been described to infect virtually all warm-blooded species including humans. Infection with the parasite occurs through the ingestion of food and water contaminated with oocysts shed in feces of infected cats or the consumption of raw and undercooked meat containing tissue cysts, or by the transplacental transmission of tachyzoites from mother to unborn child [1–4]. *Toxoplasma gondii* infection is usually asymptomatic in healthy individuals, but in immunodeficient individuals, such as unborn fetuses or patients with acquired immune deficiency syndrome (AIDS), *T. gondii* infection may cause symptomatic disease, including encephalitis, retinochoroiditis, foetus abortion, splenomegaly and pneumonitis [5, 6]. *Toxoplasma gondii* infection can also cause severe damage to livestock, inducing the acute onset of toxoplasmosis and death in pigs and abortion in sheep [1, 7]. Variation in clinical presentation and severity of disease might be attributed to the genetic heterogeneity of the host and the genotype of the infective parasite [4–6], but it remains poorly understood just how different *T. gondii* strains influence disease. The mechanisms by which *T. gondii* persists are poorly studied in pigs.

MicroRNAs (miRNAs) are 18- to 22-nucleotide-long non-coding RNAs that act on post-transcriptional gene regulation, causing degradation of messenger RNA (mRNA) or inhibiting its translation into proteins [8, 9]. miRNAs influence many cellular processes, including cell proliferation, differentiation, migration, apoptosis and angiogenesis, as well as carcinogenesis [9]. *Toxoplasma gondii* infection dysregulates the expression of specific host miRNAs, which contributes to efficient parasite replication by altering the signalling pathways involved in the defensive response of infected cells [10–13]. In our previous studies, we found that the atypical genotype Chinese I (ToxoDB#9) strain can cause acute disease in piglets and alterations in miRNA expression profiles in spleen and brain tissues, with the expression of miR-185 identified as being upregulated [14, 15].

MiR-185 is located at chromosome 22q11.21 and is haplo-insufficient in patients with 22q11.2/DiGeorge syndrome [16] who can have thymic hypoplasia, hypoparathyroidism, cardiac anomalies and/or learning disabilities [17]. Dysregulation of miR-185 mainly occurs in various pathological states, especially in human cancers. The majority of experimental data indicates that miR-185 exhibits tumour-suppressive activities by affecting many critical biological processes, such as the cell cycle, epithelial-to-mesenchymal transition, apoptosis, autophagy, invasion and metastasis [18]. In addition, miR-185 plays a crucial role in regulating bone formation and remodelling. MiR-185-5p serves as an important regulator of amelogenesis and osteoblast differentiation and may contribute to ameloporosis [19, 20]. miR-185 targets multiple genes or pathways to execute its function [18]. In our previous studies, bioinformatics analysis revealed that the A-rapidly accelerated fibrosarcoma (ARAF) gene was a target of miR-185 [14, 15].

ARAF is a serine/threonine protein kinase and a member of the Raf family protein kinases, which consist of the ARAF, BRAF and CRAF isoforms [21]; these kinases regulate a variety of basic cellular functions including proliferation, differentiation, transformation, apoptosis and metabolism, mainly through the MEK/ERK pathway [22]. In comparison with BRAF and CRAF, ARAF has the lowest basal kinase activity for activating MEK [23], with the maximal activity of ARAF being only 20% of that of CRAF [21]. ARAF is considered to be a crucial signaling hub controlling cellular processes such as proliferation, apoptosis and glucose metabolism [24]. A previous study showed that 27 and 15 out of 132 proteins interacting with ARAF were involved in apoptosis and apoptosis signal transduction, respectively [25]. ARAF has also been demonstrated to play an important role in several tumours and diseases [21]. However, to date, the role of ARAF during *T. gondii* infection has not been reported.

The aim of the present study was to confirm the regulation of miR-185 on ARAF expression and the role of the miR-185/ARAF axis in modulating host cell apoptosis during *T. gondii* infection. Our study will provide novel insights into the role of miR-185 in the host response to *T. gondii* infection, and will further deepen our understanding of the interactions between the host and *T. gondii*.

## Methods

### *Toxoplasma gondii* strains, cells and animals

The *T. gondii* YZ-1 strain, isolated from a home-bred wild boar in Jiangsu Province of China, was identified as ToxoDB #9 (Chinese I genotype), the most prevalent genotype in China, and shown to be virulent in mice in our previous study [26]. *Toxoplasma gondii* RH strain, an international standard virulent strain, was provided by Yonghua Zhou from the Jiangsu Institute of Parasitic Diseases (Wuxi, China) and maintained in our laboratory.

The PK-15 cell line was purchased from Shanghai Zhong Qiao Xin Zhou Biotechnology Co., Ltd (Shanghai, China). The cells were cultured at 37 °C with 5% CO_2_ in DMEM medium (Gibco, Shanghai, China) with 10% fetal bovine serum (FBS; Eallbio, Beijing, China) containing 100 IU/ml penicillin and 100 μg/ml streptomycin (Beyotime, Shanghai, China). The 3D4/21 cell line was maintained in our laboratory. The cells were cultured at 37 °C with 5% CO_2_ in RPMI 1640 medium (Gibco) with 10% FBS containing 100 IU/ml penicillin and 100 μg/ml streptomycin.

Specific pathogen-free (SPF) ICR mice, each weighing 22 ± 2 g, were purchased from the Comparative Medicine Center of Yangzhou University (Yangzhou, China). Mice were given free access to granulated food and water. All mice were treated in strict accordance with the recommendations of the guide for the conduct of studies with experimental animals of the People’s Republic of China. The study protocol was approved by the Animal Care and Use Committee of the College of Veterinary Medicine, Yangzhou University (Approval ID: SYXK [Su] 2012-0029).

### Vectors, primers and miRNA

The psiCHECK2 luciferase reporter vector was purchased from General Biol (Anhui, China). LentiCRISPRv2 plasmid was generously provided by Prof. Ye J (The College of Veterinary Medicine, Yangzhou University, Yangzhou, China). The pcDNA3.1-3xflag-C vector was purchased from Bast Biology (Nanjing, China). All primers (Table 1) were synthesised by Tsingke Biotechnology (Beijing, China). The miR-185 mimic and mimic control were synthesised by Suzhou Genepharma Co., Ltd (Suzhou, China), of which the miR-185 mimic was predicted to target the ARAF gene, and the mimic control was a random sequence that has no homology with any mammalian sequence (Table 1).

**Table 1:** Sequences of microRNA and primers used in the experiment

Name	Sequences (5′ to 3′)
sgRNA-F	CACCGGCCCAACAAGCAGCGCACGG
sgRNA-R	AAACCCGTGCGCTGCTTGTTGGGC*C*
Araf-F(*Eco*RI)	CCGGAATTCATGGAGCCACCACGGGGC
Araf-R(*Xho*I)	CCGCTCGAGCTAAGGCACAAGGAGGGCT
qPCR-Araf-F-pig	CAACACTGATGCTGCTGGTAA
qPCR-Araf-R-pig	CAGATGGCGACTTGGAATG
qPCR-GAPDH-F-pig	GTCGGTTGTGGATCTGACCT
qPCR-GAPDH-R-pig	CTTGACGAAGTGGTCGTTGA
qPCR-Araf-F-mice	CTCCACATCTACTCCTAACG
qPCR-Araf-R-mice	CCATCACCACCTCTACCA
qPCR-GAPDH-F-mice	TCTCCTGCGACTTCAACA
qPCR-GAPDH-R-mice	TGTAGCCGTATTCATTGTCA
qPCR-miR-185	TGGAGAGAAAGGCAGTTCCTGA
Detection-sgRNA-F	TTGACAAAGTCCTAGGCTCCAT
Detection-sgRNA-R	CACCTTTCTGATCCACGATGT
PsiCHECK2 (*Xho* I)-ARAF-F-P1	GCCTCGAGGCCCCACCCCCAGCCACC
Araf-M-R-P2	GATGGAGGAGCCGGACTATTTATAAGTATCCCCAAAG
Araf-M-R-P3	TACTTATAAATAGTCCGGCTCCTCCATCTCCAATGGC
PsiCHECK2 (*Not* I)-ARAF-F-P4	CGGCGGCCGCCCAGCAGAATTCTGTGCTT
*miR-185 mimic*	
Sense	UGGAGAGAAAGGCAGUUCCUGA
Antisense	AGGAACUGCCUUUCUCUCCAUU
*Mimic control*	
Sense	UUCUCCGAACGUGUCACGUTT
Antisense	ACGUGACACGUUCGGAGAATT

Restriction enzyme cutting sites are shown in underlining and italics

### Quantitative real-time PCR

Total RNA was isolated from PK-15 cells, 3D4/21 cells and mouse viscera using the FastPure Cell/Tissue Total RNA Isolation Kit V2 (Vazyme, Nanjing, China) according to the manufacturer’s protocol. The mRNA was reverse transcribed using the HiScript III RT SuperMix for qPCR Kit (Vazyme) and Mir-X miRNA First-Strand Synthesis Kit (Takara, Beijing, China) according to the respective manufacturer’s protocol. Quantitative real-time PCR (qPCR) reactions were performed using the AceQ Universal SYBR qPCR Master Mix (Vazyme) in a 20-μl reaction mixture; each reaction was performed in triplicate. The expression levels of the ARAF gene were quantified and normalised to glyceraldehyde 3-phosphate dehydrogenase (GAPDH) using the 2^−ΔΔCt^ method, and the expression levels of miR-185 were quantified and normalised to U6 (Takara) using the 2^−ΔΔCt^ method.

### Western blot

Cellular proteins from PK-15 and 3D4/21 cells were extracted using RIPA Lysis Buffer (Takara) supplemented with proteinase inhibitor cocktail (Beyotime). The protein concentrations were determined using the BCA Protein Assay Kit (Beyotime). Equal amounts of proteins were subjected to 10–15% sodium dodecyl sulfate–polyacrylamide gel electrophoresis (SDS-PAGE), and the products subsequently transferred onto nitrocellulose membranes. The membranes were then blocked with nonfat milk for 2 h, incubated with the primary antibody overnight at 4 °C and then washed twice with TBST, following which the membranes were incubated with the secondary antibodies for 1 h at room temperature. To detect ARAF, its epitopes were detected using rat anti-ARAF antibody (Abcam, Wuhan, China) at a 1:1 000 dilution followed by secondary probing with HRP Goat Anti-Rabbit IgG (H + L) (ABclonal, Wuhan, China) at a 1:10 000 dilution. To detect cleaved-caspase-3, its epitopes were detected using rat anti-cleaved-caspase-3 antibody (ImmunoWay, SuZhou, China) at a 1:1000 dilution, followed by secondary probing with HRP Goat Anti-Rabbit IgG (H + L) at a 1:10,000 dilution. Protein bands were detected using the ECL Detection Kit (Tanon, Beijing, China) and imaged.

To ensure equal loading of samples, we also probed with anti-GAPDH Mouse monoclonal antibody (mAb; ABclonal) at a 1:5000 dilution followed by HRP Goat Anti-Mouse IgG (ABclonal) at a 1:10,000 dilution.

### Detection of expression level of miR-185 and ARAF after* T. gondii* infection

#### Experiment in vivo

Nine mice were randomly assigned to three groups of three mice each. The mice were intraperitoneally infected with 200 tachyzoites of *T. gondii* RH strain or 2000 tachyzoites of *T. gondii* YZ-1 strain, respectively. At 10 days post-challenge, the mice were sacrificed and their liver, spleen, heart, lung and kidney tissues were separately removed. Total RNA and proteins were extracted from each of these tissues using the FastPure Cell/Tissue Total RNA Isolation Kit V2 (Vazyme). The expression levels of miR-185 and ARAF gene were detected by qPCR. The uninfected mouse tissues served as the negative control.

#### Experiment in vitro

PK-15 and 3D4/21 cells were plated in 6-well plates 1 day in advance of the experiment. When the cell density had increased to 60%–80%, PK-15 and 3D4/21 cells were inoculated with tachyzoites of *T. gondii* YZ-1 strain or *T. gondii* RH strain, respectively, at a parasite to cell ratio of 5:1. At 6 h after inoculation, the medium was changed to DMEM or DMEM/1640 containing 2% FBS. At 24 h of infection, the cells were collected for RNA and protein extraction. The expression levels of miR-185 and ARAF gene were detected by qPCR and Western blot, respectively. The groups without inoculation were used as blank controls. The test was repeated three times.

### Detection of expression level of miR-185 and ARAF after miRNA transfection

PK-15 and 3D4/21 cells were plated in 6-well plates 1 day in advance of the experiment. When the cell density had increased to 60–80%, cells were transfected in accordance with the guidelines of Lipofectamine RNAiMAX (Invitrogen Shanghai, China). Briefly, a total of 150 µl non-serum-supplemented Opti-MEN (Invitrogen) was used to dilute 9 µl Lipofectamine RNAiMAX or 100 pmol miR-185 mimics (the final concentration: 50 nM), respectively. Then the two types of liquid were mixed, incubated at room temperature for 5 min and then placed into culture plates. After culture at 37 °C with 5% CO_2_ for 8 h, the cells were transferred into complete medium for 24 h and then collected for detecting the expression levels of miR-185 and ARAF by qPCR and Western blot. The cells transfected with mimic control were used as the blank control. The test was repeated three times.

### Dual-luciferase reporter assay

The relationship between ARAF and miR-185 was verified by the dual luciferase reporter gene assay. Briefly, the potential binding sites of MiR-185 to ARAF was predicted by RNAhybrid software (https://bibiserv.cebitec.uni-bielefeld.de/rnahybrid). The original fragment of ARAF was cloned with primer PsiCHECK2 (*Xho*I)-ARAF-F-P1 and PsiCHECK2 (*Not*I)-Aarf-F-P4. ARAF-M-R-P2 and ARAF-M-R-P3 primers containing mutation sites were designed as described by Ho et al. [27], and then the fragment of ARAF containing the mutated site was amplified and sequenced. The original fragment and mutation fragment were cloned to the luciferase gene downstream of psiCHECK2. The recombinant vectors were named psiCHECK2-ARAF WT and psiCHECK2-ARAF MUT, respectively, and used for the experiment in PK-15 cells. In this experiment, the cells were divided into three groups: NC (cells transfected with psiCHECK2), ARAF WT (cells transfected with psiCHECK2-ARAF WT), and ARAF MUT (cells transfected with psiCHECK2-ARAF MUT), and each group was co-transfected with miR-185 mimic or mimic control, respectively. Cells were co-transfected with vectors and miR-185 mimic or mimic control using Lipofectamine 3000 (Invitrogen) and then cultured as described above. Finally, Firefly and Renilla luciferase activities were measured using the Dual-Luciferase Reporter Assay System (Promega, Beijing, China) according to the manufacturer’s instructions. The test was repeated three times.

### Detection of apoptosis in cells transfected with miR-185 mimic and infected with *T. gondii*

To investigate the roles of miR-185 in regulating apoptosis during *T. gondii* infection, PK-15 and 3D4/21 cells were each divided into four groups: (i) a group transfected with miR-185 mimic/*T. gondii* infection; (ii) a group transfected with miR-185 mimic/without *T. gondii* infection; (iii) a group transfected with miR-185 mimic control/*T. gondii* infection; and (iv) a group transfected with miR-185 mimic control/without *T. gondii* infection. Cells were transfected with miRNA by Lipofectamine RNAiMAX and were inoculated with *T. gondii* YZ-1 strain as described above. At 24 h after inoculation, the cells were harvested and stained by the Annexin V-FITC/PI Apoptosis Detection Kit (Vazyme), and apoptosis was evaluated using a CytoFLEX Flow Cytometer (Beckman, Shanghai, China). The protein expression levels of cleaved caspase-3 (CL-caspase3), which is a key pro-apoptotic protein involved in the execution phase of multiple apoptotic pathways, was detected by Western blot.

### Establishment of cell lines with stable knockout of the ARAF gene

According to the sequence of the ARAF gene, the single guide RNA (sgRNA) was designed by CHOPCHOP software (http://chopchop.cbu.uib.no/), and a pair of primers named sgRNA-F and sgRNA-R (Table1) were synthesised. According to the manufacturer’s instructions, the sgRNA was annealed to form double-stranded DNA and then cloned into the lentiCRISPRv2 vector and named lentiCRISPRv2-KOARAF. PK-15 and 3D4/21 cells were transfected with lentiCRISPRv2-KOARAF plasmid using Lipofectamine 3000 as described above, and selected by puromycin. The cell lines (named PK-15-KOARAF and 3D4/21-KOARAF cells) with stable knockout of the ARAF gene were selected by subcloning, and ARAF knockout was verified by qPCR, Western blot and sequencing.

### Construction of ARAF overexpression vector

The overexpression vector of ARAF was constructed as previously described [28]. Briefly, the full-length coding sequences (CDS) of ARAF were amplified by PCR with primers ARAF-F and ARAF-R (Table 1). PCR products were ligated into pcDNA3.1-3xflag-C vector. The vector containing the full-length CDS was named pcDNA3.1-Flag-ARAF; the vector without the ARAF fragment was named pcDNA3.1-Flag. As described above, the recombinant vector pcDNA3.1-Flag-ARAF was transfected into PK-15, PK-15-KOARAF, 3D4/21 and 3D4/21-KOARAF cells, and 24 h after transfection, the efficiency of ARAF overexpression was evaluated by Western blot. The cells transfected with pcDNA3.1-Flag were used as blank controls. The test was repeated three times.

### Detection of apoptosis in ARAF knockout cells infected with *T. gondii*

To explore whether ARAF is involved in regulating apoptosis during *T. gondii* infection, apoptosis rates and protein expression levels of cleaved caspase-3 were detected in PK-15, PK-15-KOARAF, 3D4/21 and 3D4/21-KOARAF cells, respectively. Briefly, each cell line was divided into two groups: (i) a group with *T. gondii* infection; and (ii) a group without *T. gondii* infection. The cells were inoculated with *T. gondii* YZ-1 strain as described above. At 24 h after inoculation, the cells were harvested and used to detect the levels of apoptosis and cleaved caspase-3 expression as described above. The test was repeated three times.

In order to further confirm that *T. gondii* infection regulate apoptosis of host cells through a miR-185-ARAF axis, the complementation test was performed in both PK-15-KOARAF and 3D4/21-KOARAF cells using ARAF overexpression vector. Briefly, each cell line was divided into four groups: (i) a group transfected with pcDNA3.1-Flag-ARAF/*T. gondii* infection; (ii) a group transfected with pcDNA3.1-Flag-ARAF/without *T. gondii* infection; (iii) a group transfected with pcDNA3.1-Flag/*T. gondii* infection; and (iv) a group transfected with pcDNA3.1-Flag/without *T. gondii* infection. The cells were transfected with vector and inoculated with *T. gondii* YZ-1 strain as described above. At 24 h after inoculation, the cells were harvested and used to detect the levels of apoptosis and cleaved caspase-3 expression as described above. The test was repeated three times.

### Statistical analysis

All statistical data presented are representative of ≥ 3 independent experiments and presented as the mean ± standard deviation (SD). Comparisons between two groups were analysed using the t-test, and comparisons among multiple groups were analyzed using one-way analysis of variance. Statistical significance was set at *P* < 0.05. All statistical analyses were carried out using GraphPad Prism 9.5 software (GraphPad Software, San Diego, CA, USA).

## Results

### *Toxoplasma gondii* infection promoting expression of miR-185 and inhibiting expression of ARAF

**Fig. 1:**
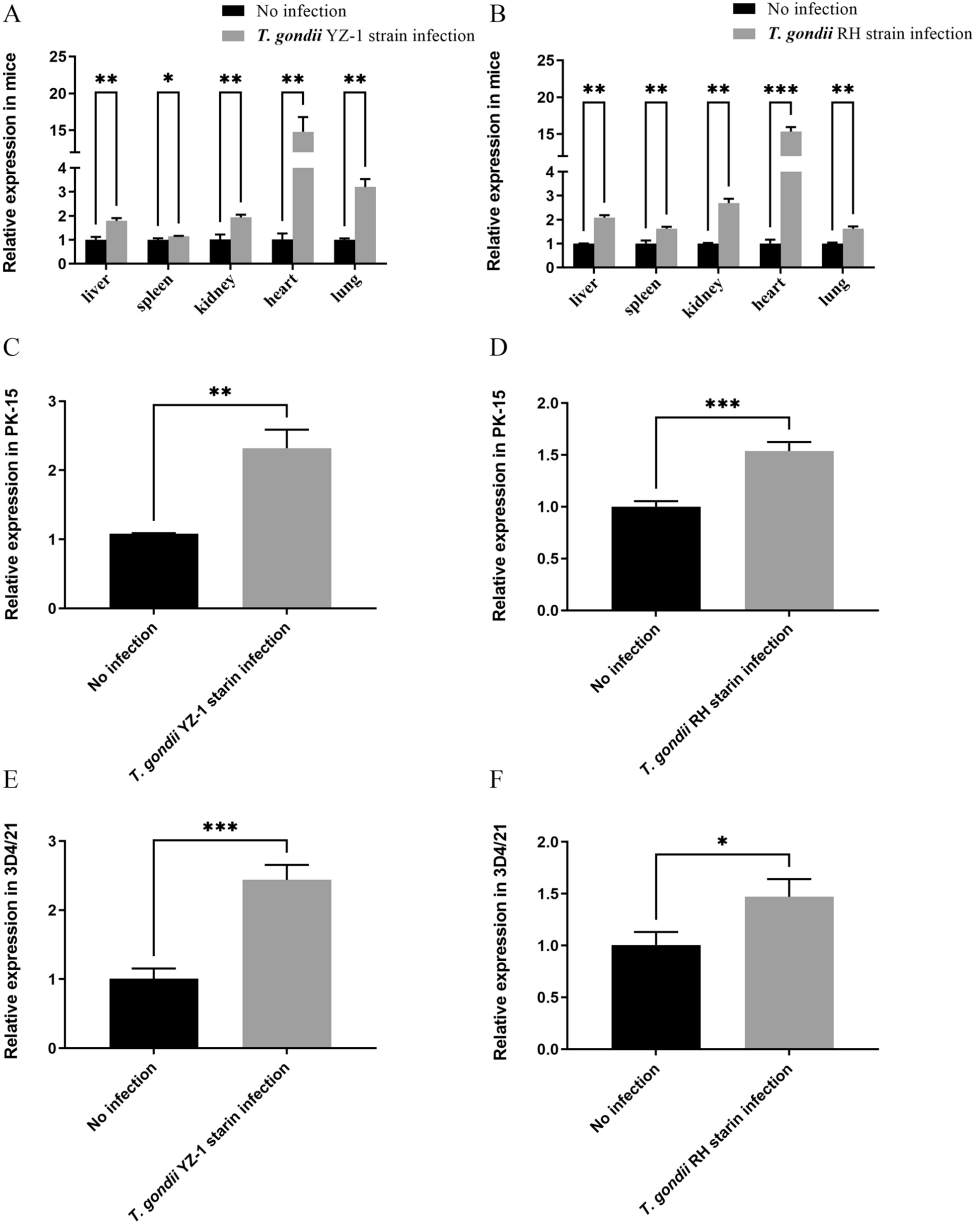
Expression levels of miR-185 after *Toxoplasma gondii* infection. **a** Quantification of miR-185 in different organs of mice with *T. gondii* YZ-1 strain infection and without infection. **b** Quantification of miR-185 in different organs of mice with *T. gondii* RH strain infection and without infection. **c** Quantification of miR-185 in PK-15 (porcine kidney epithelial) cells with *T. gondii* YZ-1 strain infection and without infection. **d** Quantification of miR-185 in PK-15 cells with *T. gondii* RH strain infection and without infection. **e** Quantification of miR-185 in 3D4/21 cells (porcine alveolar macrophages) with *T. gondii* YZ-1 strain infection and without infection. **f** Quantification of miR-185 in 3D4/21 cells with *T. gondii* RH strain infection and without infection. Asterisks indicate a significant difference between controls (un-infected cells) and infected cells at *P < 0.05, **P < 0.01 and ***P < 0.001. miR-185, MicroRNA-185

**Fig. 2:**
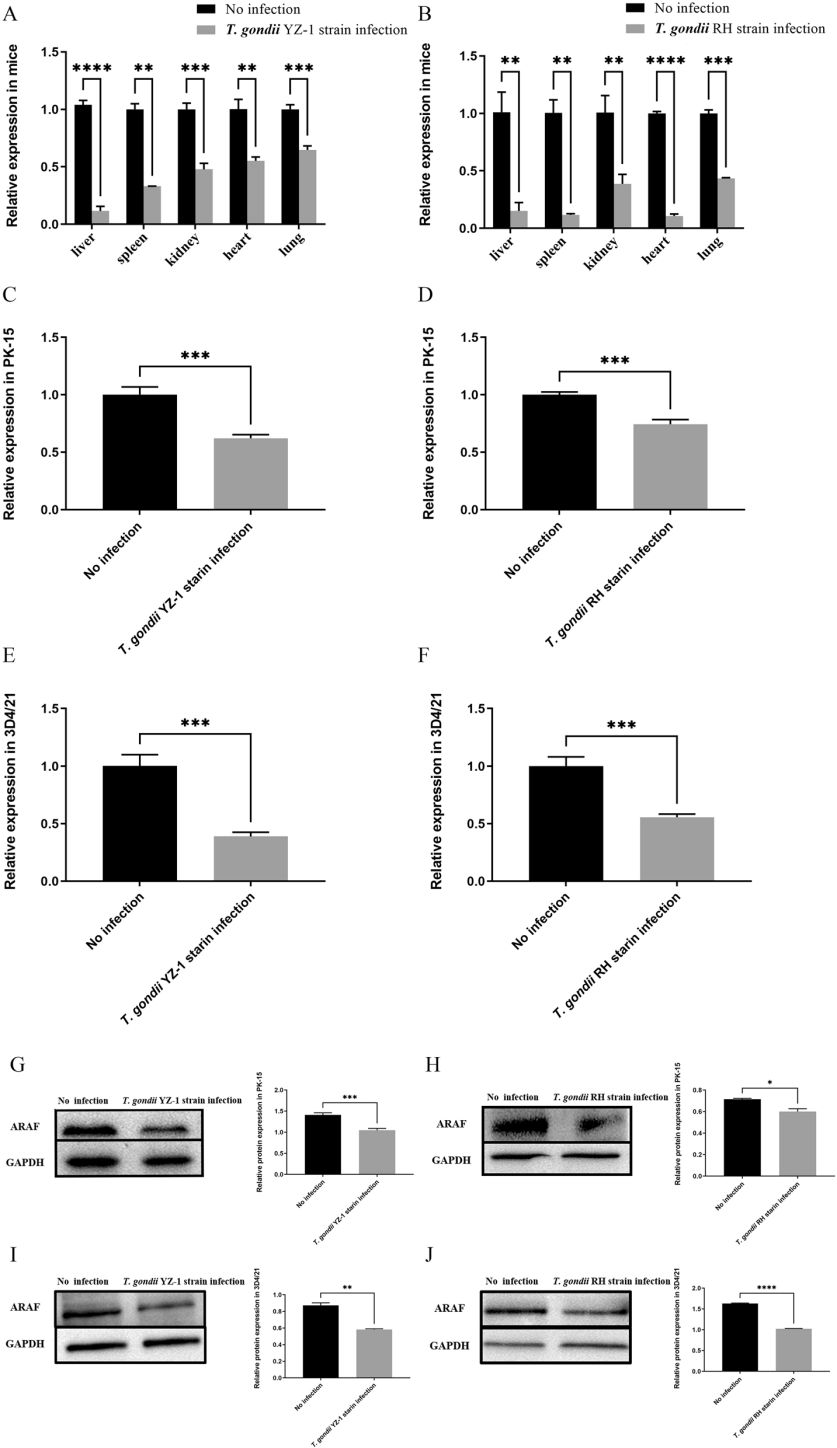
Expression levels of ARAF after *T. gondii* infection. **a** Quantification of ARAF in different organs of mice with *T. gondii* YZ-1 strain infection and without infection. **b** Quantification of ARAF in different organs of mice with *T. gondii* RH strain infection and without infection. **c** Quantification of ARAF in PK-15 (porcine kidney epithelial) cells with *T. gondii* YZ-1 strain infection and without infection. **d** Quantification of ARAF in PK-15 cells with *T. gondii* RH strain infection and without infection. **e** Quantification of ARAF in 3D4/21 cells (porcine alveolar macrophages) with *T. gondii* YZ-1 strain infection and without infection. **f** Quantification of ARAF in 3D4/21 cells with *T. gondii* RH strain infection and without infection. **g** Western blot detecting ARAF protein decrease in PK-15 cells after *T. gondii* YZ-1 strain infection; the bar graph indicates the relative protein expression of ARAF. **h** Western blot detecting ARAF protein decrease in PK-15 cells after *T. gondii* RH strain infection; the bar graph indicates the relative protein expression of ARAF. **i** Western blot detecting ARAF protein decrease in 3D4/21 cells after *T. gondii* YZ-1 strain infection; the bar graph indicates the relative protein expression of ARAF. **j** Western blot detecting ARAF protein decrease in 3D4/21 cells after *T. gondii* RH strain infection; the bar graph indicates the relative protein expression of ARAF. Asterisks indicate a significant difference between controls (un-infected cells) and infected cells at *P < 0.05, **P < 0.01, ***P < 0.001 and ****P < 0.0001

**Fig. 3:**
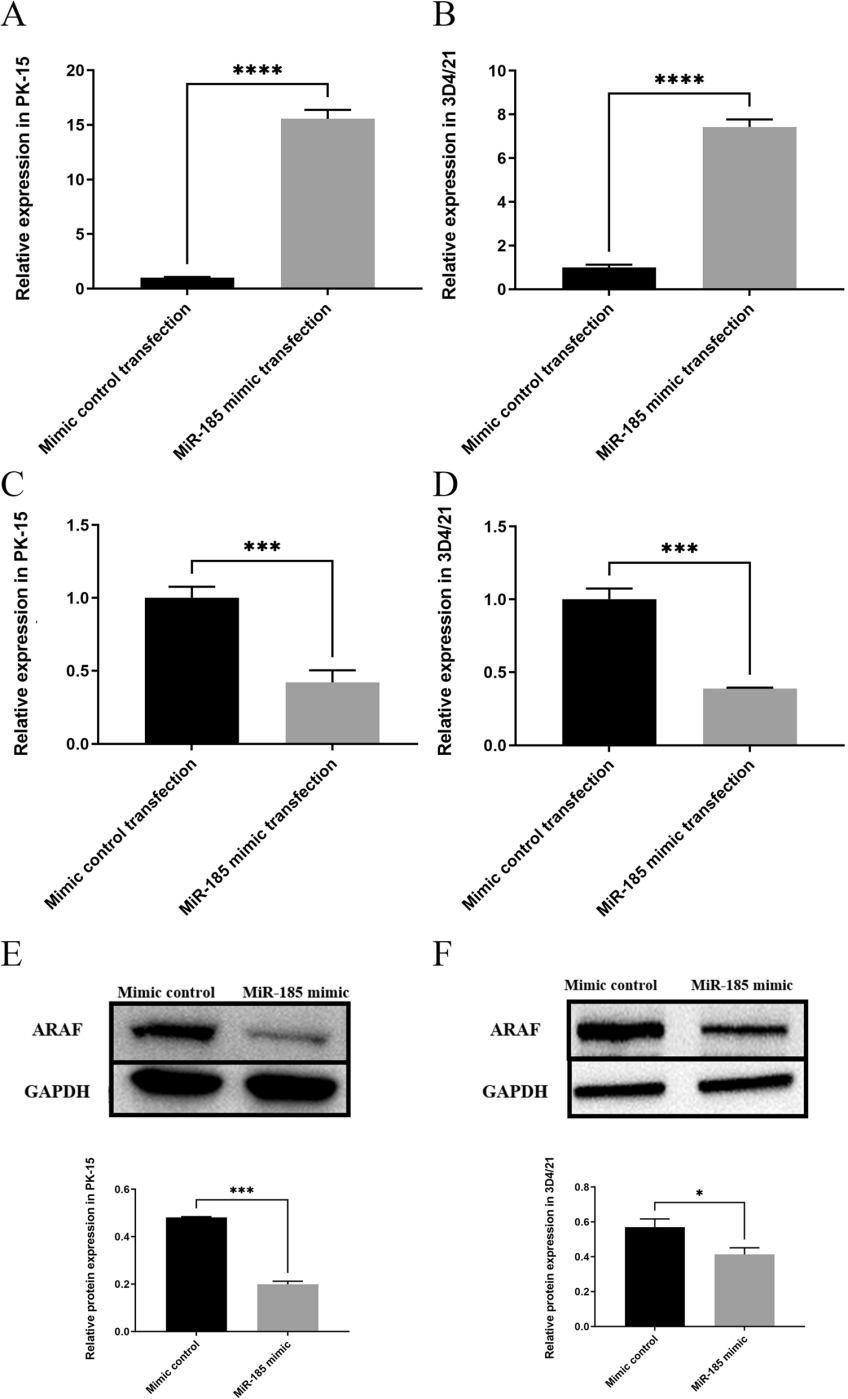
Expression levels of miR-185 and ARAF after miRNA transfection. **a** Quantification of miR-185 in PK-15 (porcine kidney epithelial) cells transfected with miR-185 mimic and mimic control. **b** Quantification of miR-185 in 3D4/21 cells (porcine alveolar macrophages) transfected with miR-185 mimic and mimic control. **c** Quantification of ARAF in PK-15 cells transfected with miR-185 mimic and mimic control. **d** Quantification of ARAF in 3D4/21 cells transfected with miR-185 mimic and mimic control. **e** Western blot detection of decrease in expression of ARAF protein after miRNA transfection in PK-15 cells; the bar graph indicates the relative protein expression of ARAF. **f** Western blot detection of decrease in ARAF protein after miRNA transfection in 3D4/21 cells; the bar graph indicates the relative protein expression of ARAF. Asterisks indicate a significant difference between cells transfected with mimic controls and cells transfected with miR-185 mimic at *P < 0.05, ***P < 0.001 and ****P < 0.0001

The levels of miR-185 and the mRNA levels of ARAF in mice and in PK-15 and 3D4/21 cells infected with *T. gondii* YZ-1 and RH strains, respectively, were determined by qPCR. The results showed that, compared with the groups with no infection, the levels of miR-185 in mouse tissues (Fig. 1a, b), PK-15 cells (Fig. 1c, d) and 3D4/21 cells (Fig. 1e, f) were significantly elevated (*t* = 3.820, *P* < 0.05), whereas the mRNA levels of ARAF in mouse tissues (Fig. 2a, b), PK-15 cells (Fig. 2c, d) and 3D4/21 cells (Fig. 2e, f) were significantly decreased (*t *= 8.563, *P* < 0.01). In contrast, the protein expression levels of ARAF in cells were detected using Western blot. The results showed that, compared with the groups without infection, the protein expression levels of ARAF in PK-15 (Fig. 2g, h) and 3D4/21 (Fig. 2i, j) cells were significantly decreased (*t* = 7.409, *P* < 0.05). These results suggest that *T. gondii* infection might promote the expression of miR-185 and inhibit the expression of ARAF both in vivo and in vitro, which occurred in both porcine macrophages (3D4/21) and epithelial cells (PK-15) infected with the Chinese I strain and RH strain *of T. gondii*, respectively.

### MiR-185 mimic transfection inhibits expression of ARAF

To confirm that the downregulation of ARAF was achieved by miR-185, qPCR was performed to detect the levels of miR-185 and the mRNA levels of ARAF in PK-15 and 3D4/21 cells transfected with mimic. The results showed that, compared with the cells transfected with the mimic control, the levels of miR-185 in cells transfected with miR-185 mimic were significantly increased (Fig. 3a, b; *t* = 31.09, *P* < 0.0001), whereas the expression levels of ARAF mRNA in cells transfected with miR-185 mimic were significantly decreased (Fig. 3c, d; *t *= 9.092, *P* < 0.001). Western blot analysis further revealed that the protein expression levels of ARAF were also decreased after transfection (Fig. 3e, f). These results suggest that ARAF may be the target mRNA for miR-185 in PK-15 and 3D4/21 cells.

### MiR-185 directly targets ARAF

**Fig. 4:**
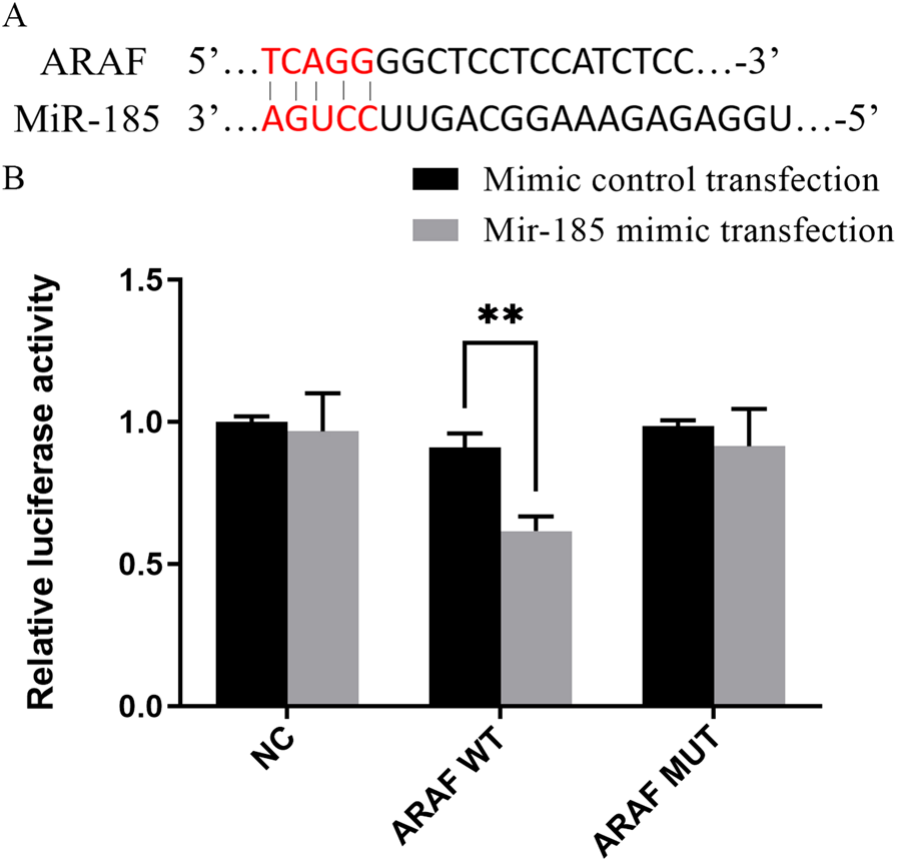
The results of the dual-luciferase reporter assay. **a** The binding site between miR-185 and ARAF gene. **b** Luciferase assay of the reporter plasmids including the wild-type (WT) and mutant (MUT) ARAF 3′-untranslated region. NC, cells transfected with psiCHECK2, and co-transfected with miR-185 mimic or mimic control, respectively; ARAF WT, cells transfected with psiCHECK2-ARAF WT, and co-transfected with miR-185 mimic or mimic control, respectively; ARAF MUT, cells transfected with psiCHECK2-ARAF MUT, and co-transfected with miR-185 mimic or mimic control, respectively. Asterisks indicate a significant difference at **P < 0.01.

To further determine whether ARAF is a direct target gene of miR-185, the miR-185 target site was identified by an in silico miRNA target prediction search, followed by a dual luciferase reporter assay in PK-15 cells. The results showed that the binding site for miR-185 was composed of five continuous base pairs in the 3′ end of the ARAF gene (Fig. 4a). The double fluorescent reporter gene system showed that miR-185 mimics clearly inhibited luciferase activity in the psiCHECK2-ARAF WT group (*t* = 7.141, *P* < 0.01; Fig. 4b), while miR-185 mimics had no significant effect on luciferase activity in the NC and psiCHECK2-ARAF MUT groups (*P* > 0.05; Fig. 4b). These results suggest that ARAF is the target gene of miR-185. Therefore, miR-185 could target the mRNA of ARAF and negatively regulate the expression of ARAF.

### MiR-185 regulating apoptosis of *T. gondii-*infected cells

**Fig. 5:**
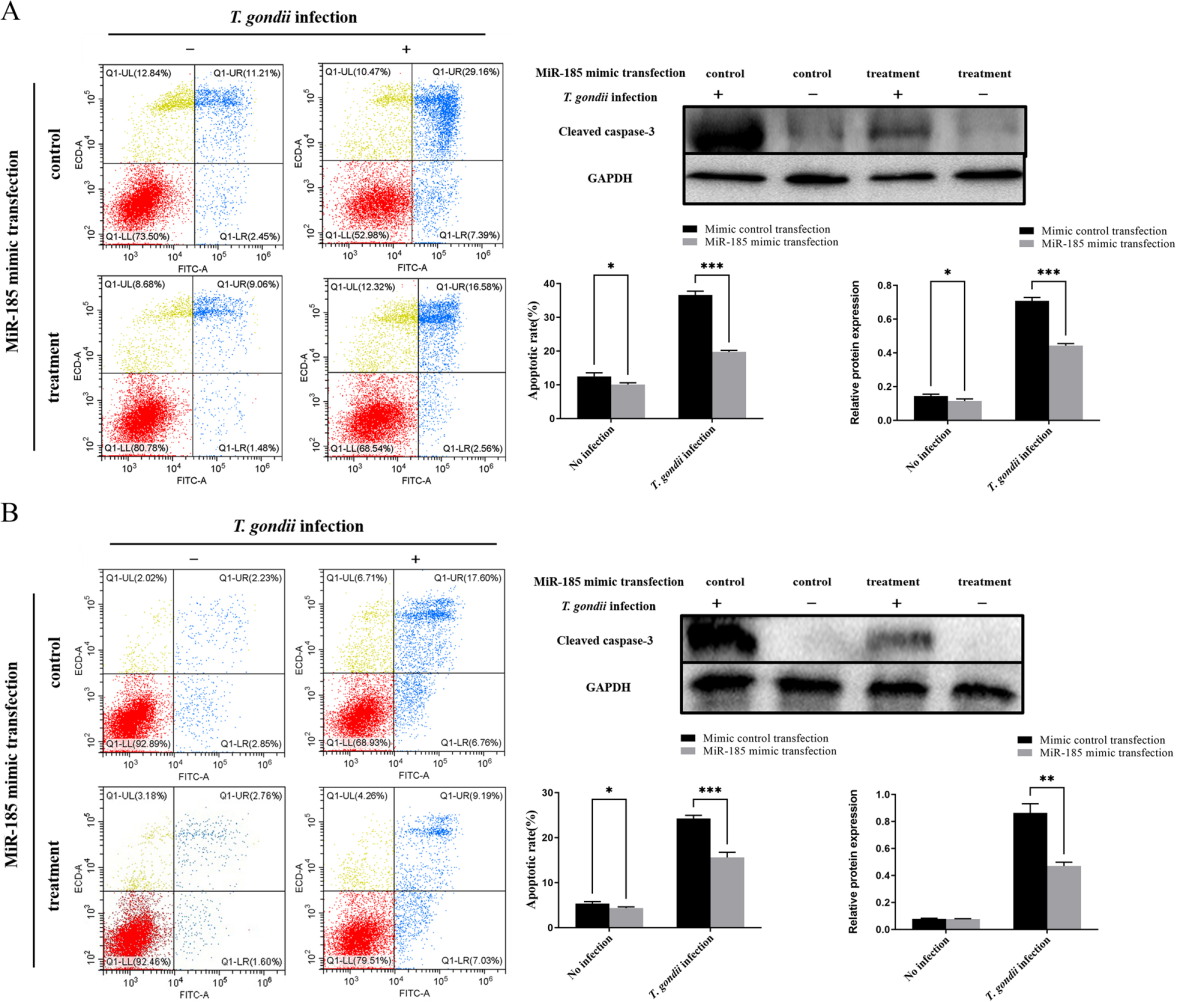
Apoptosis rates of different cells with different treatments. The apoptosis of cells was analysed by flow cytometry following staining with Annexin V and PI. Dot plots depict the amounts of cells stained with Annexin V and/or PI; numbers in parentheses indicate percentages of cells in each quadrant from a representative experiment. Western blot detecting the expression levels of cleaved caspase-3 in different cells with different treatments. The bar graphs indicate the apoptosis rates and relative protein expression of cleaved caspase-3. All cells were separately infected with *T. gondii* YZ-1 strain and control cultivation. **a** Apoptosis rates and the expression levels of cleaved caspase-3 in PK-15 (porcine kidney epithelial) cells transfected with miR-185 mimic or mimic control. **b** Apoptosis rates and the expression levels of cleaved caspase-3 in 3D4/21 cells transfected with miR-185 mimic or mimic control. Asterisk indicates a significant difference at *P < 0.05, **P < 0.01 and ***P < 0.001. PI, Propidium iodide

In order to investigate whether miR-185 is involved in regulating apoptosis of *T. gondii-*infected cells, we examined the apoptotic rates of the PK-15 and 3D4/21 cells transfected with miR-185 mimic or miR-185 mimic control, when infected or not infected with *T. gondii*. The cell apoptosis rate was determined by flow cytometry using double labelling with Annexin V-FITC and propidium iodide. The results showed that following *T. gondii* infection, the apoptotic rates of PK-15 cells transfected with mimic control were significantly higher than those of cells transfected with miR-185 mimic (*t* = 23.40, *P* < 0.001; Fig. 5a). Similarly, the results showed that following *T. gondii* infection, the apoptotic rates of 3D4/21 cells transfected with mimic control were significantly higher than those of cells transfected with miR-185 mimic (*t *= 10.87, *P* < 0.001; Fig. 5b). In addition, compared with the group transfected with miR-185 mimic/*T. gondii* infection, the protein expression levels of cleaved caspase-3 protein in the group transfected with miR-185 mimic control/*T. gondii* infection were significantly elevated (*t* = 9.242, *P* < 0.01; Fig. 5). These results suggest that miR-185 overexpression could inhibit the apoptosis of cells induced by *T. gondii*.

### Knockout efficiency and overexpression of ARAF gene in the cell lines

**Fig. 6:**
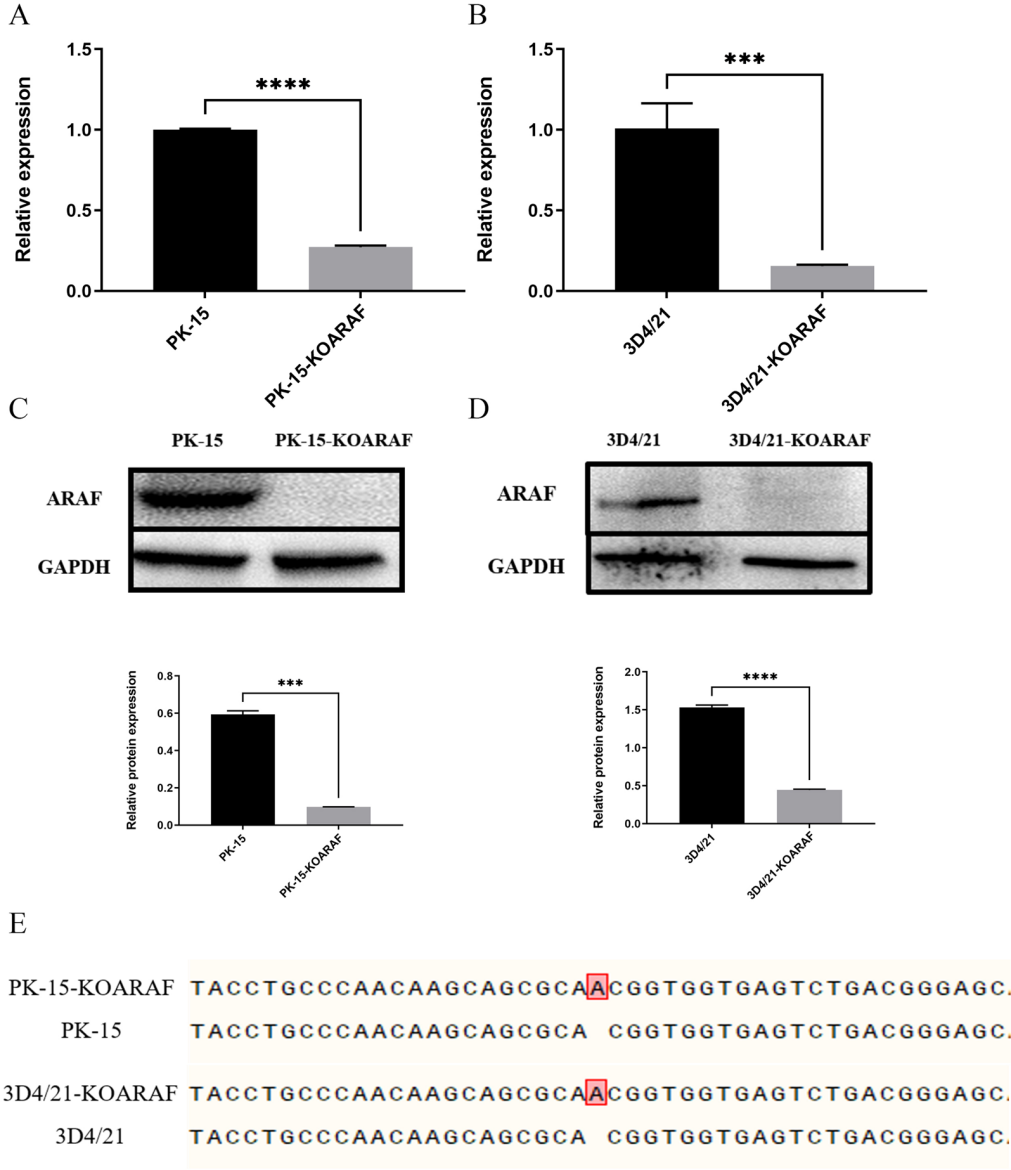
Knockout efficiency of cell lines with stable ARAF gene knockout. **a** Quantification of ARAF in PK-15-KOARAF cells and PK-15 cells. **b** Quantification of ARAF in 3D4/21-KoARAF cells and 3D4/21 cells. **c** Western blot detecting ARAF protein loss in PK-15-KOARAF cells; the bar graph indicates the relative protein expression of ARAF. **d** Western blot detecting ARAF protein loss in 3D4/21-KoARAF cells. **e** Mutation site of ARAF in PK-15-KOARAF and 3D4/21-KoARAF cells (adenine [A]); the bar graph indicates the relative protein expression of ARAF. Asterisks indicate a significant difference at ***P < 0.001 and ****P < 0.0001. 3D4/21-KoARAF, 3D4/21 cell line with stable ARAF gene knockout; GAPDH, glyceraldehyde 3-phosphate dehydrogenase; PK-15-KOARAF, PK-15 cell line with stable ARAF gene knockout

To examine whether ARAF is involved in regulating apoptosis of *T. gondii-*infected cells, both PK-15 and 3D4/21 cell lines with stable ARAF knockout were successfully constructed. qPCR assays showed that, compared to control PK-15 and 3D4/21 cells, mRNA expression levels of ARAF in cells of both of the knockout lines (PK-15-KOARAF and 3D4/21-KOARAF) were significantly downregulated (*t* = 49.29, *P* < 0.001; Fig. 6a, b). Western blot analysis also revealed that expression levels of ARAF protein in PK-15-KOARAF and 3D4/21-KOARAF cells were significantly lower than those in PK-15 and 3D4/21 cells, respectively (*t* = 43.91, *P* < 0.001; Fig. 6c, d). In addition, the result of sequencing further demonstrated that the insertion of one base (adenine [A]) resulted in frameshift mutations of the ARAF gene (Fig. 6e). These results confirmed that the ARAF gene in both PK-15-KOARAF and 3D4/21-KOARAF cells was efficiently knocked out.

**Fig. 7:**
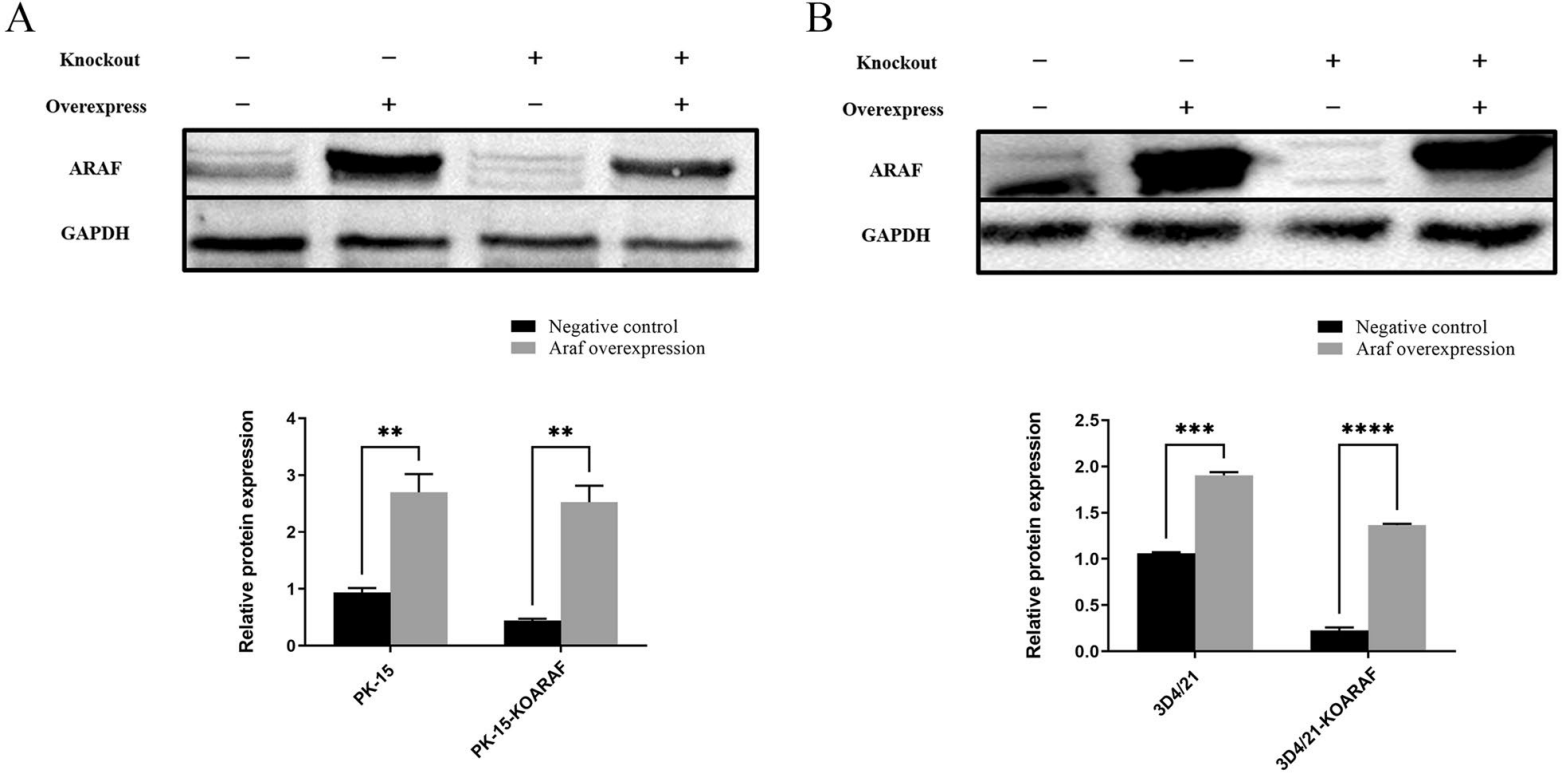
The overexpression efficiency of the different cell lines after transfection with the overexpression vector. The bar graphs indicate the relative protein expression of ARAF. **a** Western blot detecting ARAF protein overexpression in PK-15 and PK-15-KOARAF cells. **b** Western blot detecting ARAF protein overexpression in 3D4/21 and 3D4/21-KoARAF cells. Asterisks indicate a significant difference at **P < 0.01, ***P < 0.001 and ****P < 0.0001. 3D4/21-KoARAF, 3D4/21 cell line with stable ARAF gene knockout; GAPDH, glyceraldehyde 3-phosphate dehydrogenase; PK-15-KOARAF, PK-15 cell line with stable ARAF gene knockout

The overexpression efficiency of the ARAF gene was detected by Western blot. The results showed that the expression levels of ARAF protein in PK-15 and PK-15-KOARAF cells transfected with the pcDNA3.1-Flag-ARAF vector were significantly higher than those in cells transfected with the pcDNA3.1-Flag vector (Fig. 7a). Similarly, the expression levels of ARAF protein in 3D4/21 and 3D4/21-KOARAF cells transfected with the pcDNA3.1-Flag-ARAF vector were significantly higher than those in cells transfected with the pcDNA3.1-Flag vector (Fig. 7b). These results further confirmed that the ARAF gene of PK-15-KOARAF and 3D4/21-KOARAF cells was efficiently knocked out in each of these cell lines and that the overexpression efficiency of pcDNA3.1-Flag-ARAF vector was sufficiently high to perform the subsequent experiment.

### *Toxoplasma gondii* infection regulating apoptosis of host cells through a miR-185/ARAF axis

**Fig. 8:**
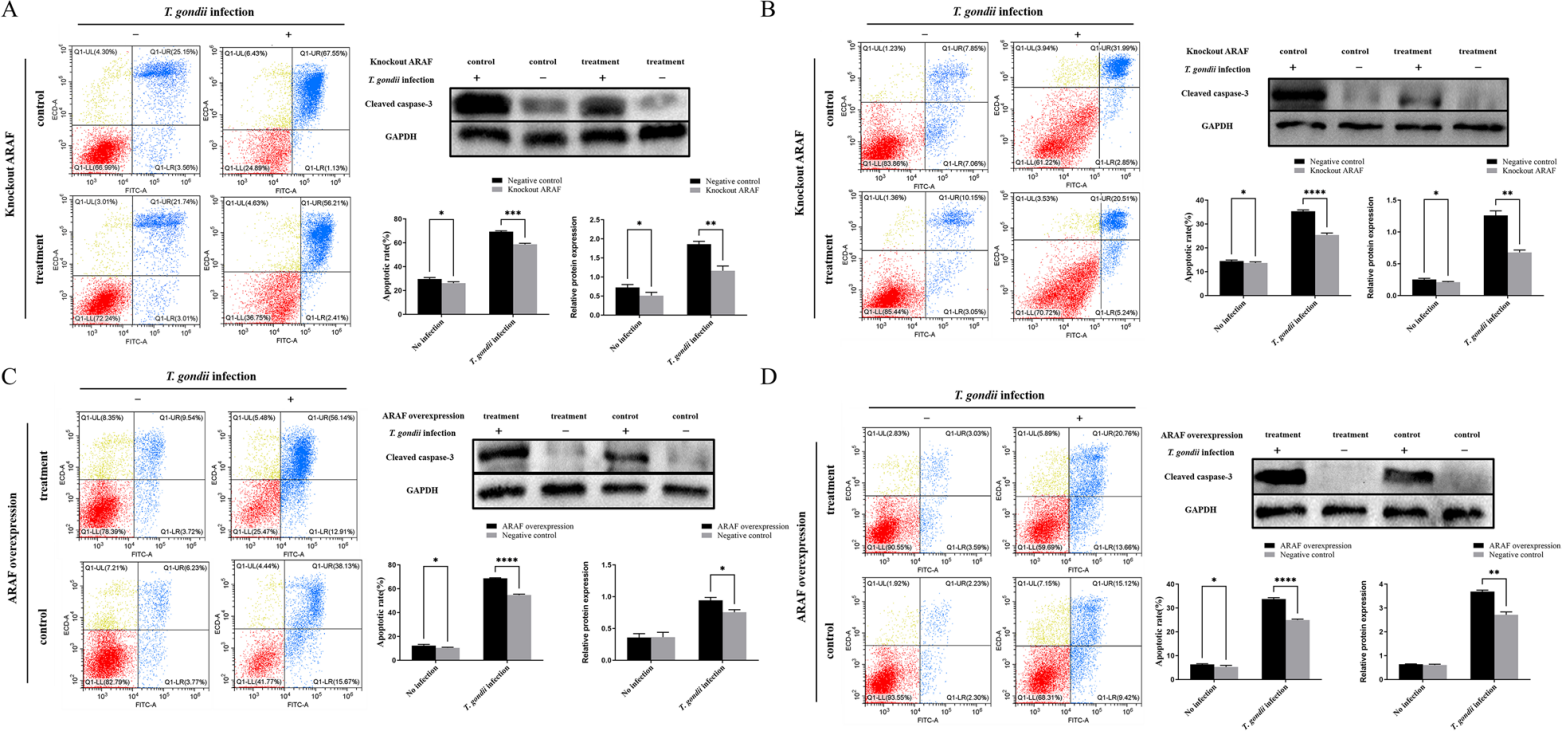
Apoptosis rates of different cells with different treatments. The apoptosis of cells was analysed by flow cytometry following staining with Annexin V and PI. Dot plots depict the amounts of cells stained with Annexin V and/or PI; numbers in parentheses indicate percentages of cells in each quadrant from a representative experiment. Western blot detecting the expression levels of cleaved caspase-3 in different cells with different treatments. The bar graphs indicate the apoptosis rates and relative protein expression of cleaved caspase-3. All cells were separately infected with *T. gondii* YZ-1 strain and control cultivation. **a** Apoptosis rates and the expression levels of cleaved caspase-3 in PK-15 cells and PK-15-KoARAF cells. **b** Apoptosis rates and the expression levels of cleaved caspase-3 in 3D4/21 cells and 3D4/21-KoARAF cells. **c** Apoptosis rates and the expression levels of cleaved caspase-3 in PK-15-KOARAF cells transfected with the overexpression vector pcDNA3.1-Flag-ARAF or pcDNA3.1-Flag. **d** Apoptosis rates and the expression levels of cleaved caspase-3 of 3D4/21-KOARAF cells transfected with the overexpression vector pcDNA3.1-Flag-ARAF or pcDNA3.1-Flag. Asterisks indicate a significant difference at *P < 0.05, **P < 0.01, ***P < 0.001 and ****P < 0.0001. D4/21-KoARAF, 3D4/21 cell line with stable ARAF gene knockout; GAPDH, glyceraldehyde 3-phosphate dehydrogenase; PI, propidium iodide; PK-15-KOARAF, PK-15 cell line with stable ARAF gene knockout

To confirm whether *T. gondii* infection regulates apoptosis of host cells through a miR-185-ARAF axis, we determined the effect of ARAF knockout on apoptosis of *T. gondii*-infected cells. The apoptosis of cells was analszed by flow cytometry following staining with Annexin V-FITC and PI. The results showed that following *T. gondii* infection, the apoptotic rates of PK-15 and 3D4/21 cells were significantly higher than those of PK-15-KOARAF and 3D4/21-KOARAF cells (*t* = 16.24, *P* < 0.001; Fig. 8a, b), respectively. We further examined the effect of complementation of ARAF knockout using the overexpression vector on apoptosis of *T. gondii*-infected cells. The results showed that following *T. gondii* infection, the apoptotic rates of PK-15-KOARAF and 3D4/21-KOARAF cells transfected with pcDNA3.1-Flag-ARAF were significantly higher than those of PK-15-KOARAF and 3D4/21-KOARAF cells transfected with pcDNA3.1-Flag, respectively (*t* = 25.59, *P* < 0.0001; Fig. 8c, d). In addition, the results showed that following *T. gondii* infection, the expression levels of cleaved caspase-3 protein in PK-15-KOARAF and 3D4/21-KOARAF cells were significantly lower than those in PK-15 and 3D4/21 cells (*t* = 8.297, *P* < 0.01; Fig. 8a, b). Similarly, the results showed that following *T. gondii* infection, the expression levels of cleaved caspase-3 protein in PK-15-KOARAF and 3D4/21-KOARAF cells transfected with pcDNA3.1-Flag were significantly lower than those in PK-15-KOARAF and 3D4/21-KOARAF cells transfected with pcDNA3.1-Flag-ARAF, respectively (*t* = 5.397, *P* < 0.05; Fig. 8c, d). These results suggest that *T. gondii* infection could regulate apoptosis of host cells via the miR-185/ARAF axis.

## Discussion

In our previous studies, we found that infection by *T. gondii* Chinese I genotype YZ-1 strain induces the upregulation of miR-185 expression in spleen and brain tissues of piglets [14, 15]. In the present study, we further confirmed this upregulation of miR-185 expression in in vivo and in vitro experiments, further demonstrating that this *T. gondii* infection-induced upregulation was not related to the strain of *T. gondii* or the host cells. We first found that *T. gondii* infection resulted in the downregulation of ARAF gene expression and revealed a negative correlation between miR-185 and ARAF in cells infected with *T. gondii*. MiR-185 overexpression could inhibit the apoptosis of cells induced by *T. gondii*.

MiRNAs regulate gene expression by interacting with the 3′-untranslated regions (UTRs) of genes. Each miRNA usually controls up to several hundred target mRNAs, while one mRNA target can be synergistically regulated by multiple miRNAs [29–31]. To date, > 80 genes and/or pathways that are targeted by miR-185 have been identified, some of which were found to be involved in regulating apoptosis of cell lines, including protein kinase b (Akt), AKT serine/threonine kinase 1 (AKT1), cell division cycle 42 (CDC42), cyclin D2 (CCND2), cyclin dependent kinase 4 (CDK4), cyclin dependent kinase 6 (CDK6), cathepsin D (CTSD), high mobility group AT-hook 2 (HMGA2) forkhead box D3 (FOXD3), ras-related gtp-binding protein 14 (RAB14), ras homolog family member A (RHOA), microphthalmia transcription factor (SMAD7), tripartite motif containing 29 (TRIM29), Akt/caspase 9 (CASP-9) pathway, Akt/nuclear factor kappa-B (NF-κB) pathway and Wnt/β-catenin pathway [18, 20, 32]. In the present study, miR-185 mimic transfection could inhibit the expression of ARAF. The dual luciferase reporter gene assay showed that the 3′-UTR region of ARAF could bind specifically to miR-185, indicating that ARAF is a novel target gene of miR-185.

Previous studies have shown that ARAF plays an important role in apoptosis, tumourigenesis and resistance to Raf inhibitors [33–35]. ARAF dysregulation is closely associated with the initiation and progression of neoplastic diseases [36]. ARAF has a strong binding ability to mammalian sterile 20-like kinase 2 (MST2) and can block the Hippo signaling pathway mediated by MST2 phosphorylation [34]. Independent of kinase activity, ARAF binds to MST2, thereby efficiently inhibiting apoptosis [34, 37]. In the present study, we found that ARAF knockout inhibited the apoptosis induced by *T. gondii* and that ARAF overexpression in knockout cells reversed these regulatory effects. These observations suggest that ARAF participates in regulation of the apoptotic process induced by *T. gondii* infection. However, the downstream molecule regulated by ARAF need further study.

MiR-185 participates in the regulation of apoptosis by targeting different genes or signaling pathways in cancer cell lines [18, 38, 39]. For example, miR-185 was found to induce apoptosis and autophagy in hepatocellular carcinoma cells by targeting different genes in the Akt signaling pathway, including AKT1, rapamycin-insensitive companion of Mtor (RICTOR) and ras homolog enriched in brain (RHEB) [40]; miR-185 directly targeted the Na + /H + exchanger isoform 1 (NHE-1) gene to inhibit endoplasmic reticulum (ER) stress-induced apoptosis for the purpose of cardiac protection [41]; miR-185 overexpression inhibited autophagy and apoptosis in dopaminergic cells from Parkinson patients by regulating the AMP-activated protein kinase (AMPK)/mechanistic target of rapamycin kinase (mTOR) signaling pathway [42]. In the present study, we observed that: (i) the upregulation of miR-185 is a specific response to *T. gondii* infection both in in vivo and in vitro, and in both somatic cells (PK-15 cell) and immune cells (3D4/21 cell); (ii) the *Toxoplasma*-induced increase of miR-185 expression is associated with a downregulation of ARAF and an increased apoptosis rate; and (iii) that *T. gondii* infection resulted in an increase of the cleaved caspase-3 protein expression. These results indicate that *T. gondii* infection could regulate apoptosis of host cells via the miR-185/ARAF axis.

Despite many studies having shown that *T. gondii* manipulates multiple pathways, including NF-κB, mitogen-activated protein kinase (MAPKinase), c-Jun-terminal kinases (JNKinase) and phosphatidylinositol 3 kinase (PI3K)/Akt, to regulate cell apoptosis [43], only a few host miRNAs have been confirmed to be involved in apoptosis pathways elaborated by *T. gondii*. Cai et al. [44] found that in human macrophages following *T. gondii* infection, the activated STAT3 binds to the promoter of the miRNA (miR-30c-1, miR-125b-2, miR-23b-27b-24–1 and miR-17~92 cluster) genes, which leads to increased miRNA expression and increased apoptosis of host cells. Further research showed that STAT3 mediates a prosurvival pathway by upregulating the miR-17–92 cluster expression that in turn targets BCL2-like 11 (Bim), leading to the survival of host cells with *Toxoplasma* infection [45]. Hence, the miR-185/ARAF axis represents an additional strategy by which *T. gondii* counteracts host-cell apoptosis in order to maintain survival and reproduce in the host cells.

In summary, our results reveal that the upregulation of miR-185 is a specific response to *T. gondii* infection both in in vivo and in vitro, and in porcine somatic cells (PK-15 cell line) and alveolar macrophages (3D4/21 cell line). ARAF is a novel target gene of miR-185. *Toxoplasma gondii* infection could regulate apoptosis of host cells via miR-185/ARAF axis, which represents an additional strategy by which *T. gondii* counteracts host-cell apoptosis in order to maintain survival and breed in the host cells.

## Data Availability

All data and materials of the experiments described here are included in this published article and its additional files.

## Abbreviations

*T. gondii*: *Toxoplasma gondii*
MiRNA: MicroRNA
ARAF: A-rapidly accelerated fibrosarcoma
QPCR: Quantitative real-time PCR
SDS-PAGE: Sodium dodecyl sulfate-polyacrylamide gel electrophoresis
*CL-caspase3 *: Cleaved caspase-3
SgRNA: Single guide RNA
